# Meaning-focused coping, pain, and affect: a diary study of hospitalized women with rheumatoid arthritis

**DOI:** 10.1007/s11136-015-1031-6

**Published:** 2015-06-06

**Authors:** Ewa Gruszczyńska, Nina Knoll

**Affiliations:** Health Psychology Department, University of Social Sciences and Humanities, Chodakowska 19/31, 03-815 Warsaw, Poland; Division of Health Psychology, Freie Universität Berlin, Habelschwerdter Allee 45, 14195 Berlin, Germany

**Keywords:** Pain, Coping, Affect, Rheumatoid arthritis, Diary study, Multilevel modeling

## Abstract

**Purpose:**

The aim of the study was to investigate the relationship between affective state, pain, and coping in hospitalized women with rheumatoid arthritis, including both between- and within-person perspectives.

**Methods:**

Participants were 95 female patients between 24 and 82 years of age (*M* = 50.91; SD = 13.80). For three consecutive days, they rated each night their state affect (positive and negative), pain level, and coping strategies (emotion-, problem- and meaning-focused ones). Relations among variables were tested with a multilevel approach with time included as a covariate.

**Results:**

Within-person meaning-focused coping suppressed the negative pain effect on emotional state, but only for positive affect (Sobel’s *z* = 2.07, *p* = .04). Moderators of the pain–affect relationship were between-person differences in pain level (*B* = −.23, SE = .08, *t* = −2.884, *p* = .004) and in meaning-focused coping (*B* = −.63, SE = .20, *t* = −2.097, *p* = .04). Specifically, suppression was significant only for patients who reported lower than sample average pain levels and for patients who reported lower than sample average use of meaning-focused strategies.

**Conclusions:**

Findings indicated that meaning-focused coping can be a crucial strategy for keeping daily positive affect in the face of chronic pain and how this effect is modified by interindividual differences. Even if restricted to the specific context, it may inform an intervention for hospitalized women with rheumatoid arthritis.

## Introduction

Rheumatoid arthritis (RA) is a chronic systemic inflammatory disease [1]. The population prevalence of RA is relatively stable and ranges between .5 and 1 % with a higher incidence rate for women than for men [2–5]. Pain is among the most serious and disabling symptoms reported by patients. It is also believed to be a crucial determinant of patients’ emotional state [6] and overall quality of life [7]. Nonetheless, empirical evidence has shown that coping strategies can qualify effects of pain on daily affect [8], especially when pain intensity ranges between low to moderate. Effects of pain-related coping strategies can be distinguished depending on their problem- or emotion-focused character. Problem-focused coping strategies are mainly related to better adjustment, whereas emotion-focused strategies were shown to be associated with higher pain and worse well-being [9–11].

However, a great majority of these studies have concentrated only on the negative side of affective well-being, and thus less is known about strategies that may create, maintain, or support positive affective states when coping with chronic pain. They can be analyzed within the scope of meaning-focused coping, which has been defined by Folkman and Park as appraisal-based efforts to derive meaning from the stressful experience in order to sustain well-being in spite of difficult times [12–14]. Positive reappraisal is at the core of meaning-focused strategies, but their functions go beyond it, including also strategies that allow to actively control the situation, create positive sensory events, or fill daily routine with meaning [12]. In addition to problem-focused and emotion-focused coping, meaning-focused coping is thus another major coping function [15, 16]. This was also supported by findings from structural analyses of different coping questionnaires (see for instance [17, 18] or [19]).

As a driving force for positive emotions under stress, meaning-focused coping may be an important part of accommodative coping [20], required when there are hardly any possibilities for major changes in objective characteristics of the situation. Growing empirical evidence has supported this assumption in the context of chronic health stressors [21–24], which suggests that meaning-focused coping may also be beneficial when dealing with chronic pain. On the basis of this knowledge, it can be supposed that creation, maintenance, or support of positive affective states when facing chronic pain is achieved through different pathways [25]. Among cognitive ones, positive reappraisal is best recognized and proved to be effective [26], especially when perceived control is low [27], which can be contrasted with the well-documented debilitating role of catastrophizing [28]. Other meaning-focused strategies, being a mixture of cognitive processing and behavioral actions, just like intentionally creating and inducing positive sensory events with special meaning (e.g., having dinner with friends, see: [12]), still require more systematic research as they are either poorly represented in existing coping questionnaires or classified within the same category as behavioral distraction. Also, meaning-focused coping with pain has not been studied yet in a day-to-day fashion.

Daily pain was shown to be associated with higher negative and lower positive affect [29]. There is an ongoing debate in the literature whether positive and negative affects are two independent dimensions [30] or two poles of one bipolar dimension [31]. However, there is an agreement that even when analyzed in the chronic pain context [32], distinct coping efforts are probably required for effective regulation of positive and negative emotional states [33–35].

Thus, it is hypothesized that higher daily intensity of meaning-focused coping correlates with a higher level of daily positive affect, but not with a lower level of daily negative affect (*hypothesis 1).* If such relations are to illustrate functional specificity of meaning-focused coping, they should be observed even after control for pain level, emotion- and problem-focused coping strategies, and interindividual variability in coping.

When looking for a possible mechanism of the relation between pain, coping, and affect, a mediation model is theoretically justified. It is also in accordance with both Folkman’s [12] and Park’s [36] view on meaning-making processes under stress. Meaning-focused strategies are a response to distress, so they can be positively correlated with pain. In that light, on more painful days, higher intensity of meaning-focused coping should be observed, which would in turn be associated with increases in positive affect, but not necessarily with decreases in negative affect (*hypothesis 2*). This way, meaning-focused coping can suppress the debilitating influence of pain on emotional state (for the detailed description of suppression see [37]). To prove such a specific effect, it should be present in a multiple mediation model [38], when adjusted for possible meditational effects of other coping strategies, i.e., problem- and emotion-focused ones.

## Methods

### Participants

The final sample consisted of 95 women hospitalized due to RA, which amounted to a response rate of 83 % of 114 patients initially asked to participate. Basic characteristics of the sample are presented in Table 1. Participants were between 24 and 82 years of age (*M* = 50.91; SD = 13.80, normal distribution: *z* K–S = .09, *df* = 95, *p* = .20). The majority of them were married or cohabiting with a partner (70.5 %) and had at least 12 years of education (80 %). They were diagnosed with RA from about 1–42 years ago (*M* = 11.23; SD = 10.24). Mean number of previous hospitalizations because of RA was 4.29 (SD = 5.36, range 0–30); for 3.2 % it was the first hospitalization. Exacerbation of disease was the major cause for current hospitalizations. All diagnoses of RA were confirmed by a physician. Eighty-three percent of participants reported taking analgesic medication during time of the study.

**Table 1:** Demographic and clinical characteristics of participants (*N* = 95)

Variable	*N* (%)
*Age in years* (M ± SD)	50.91 ± 13.80
*Age range in years*	
24–33	13 (13.7)
34–43	16 (16.8)
44–53	23 (24.2)
54–63	23 (24.2)
64–73	15 (15.8)
74–82	5 (5.3)
*Marital status*	
Married/cohabited	67 (70.5)
Single	28 (29.5)
*Education*	
Elementary school education	7 (7.4)
Basic vocational education	12 (12.6)
High school education	44 (46.3)
University education	32 (33.7)
*Time since diagnosis* * in years* (*M* ± SD)	11.34 ± 10.24
*Range of time since diagnosis in years*	
0.5–10	53 (55.85)
11–20	23 (24.2)
21–30	15 (15.8)
31–40	3 (3.2)
41–42	1 (1.0)
*Number of previous hospitalizations due to RA*	
0	3 (3.2)
1	29 (30.5)
2	14 (14.7)
3	9 (9.5)
4 and more	36 (42.7)
Missing data	9 (9.5)
*Number of disease flares*	
1	17 (17.9)
2	19 (20)
3	4 (4.2)
4	11 (11.6)
5	16 (16.8)
6	19 (20)
7	9 (9.5)
*Past surgical intervention due to RA*	
Yes	31 (32.6)
No	65 (68.4)
*Classification of global functional status * [72]	
Class I. Completely able to perform usual activities of daily living (self-care, vocational, and avocational)	4 (4.2)
Class II. Able to perform usual self-care and vocational activities, but limited in avocational activities	29 (30.5)
Class III. Able to perform usual self-care activities, but limited in vocational and avocational activities	48 (50.5)
Class IV. Limited in ability to perform usual self-care, vocational, and avocational activities	13 (13.7)
Missing data	1 (1.1)
*Intake of analgesic medication during time of the study*	
Yes	79 (83.2)
No	16 (16.8)

*Note* *M* mean, *SD* standard deviation

### Procedure

The study was approved by the institutional ethics committee. Inclusion criteria were as follows: being female, at least 18 years old, with a confirmed diagnosis of RA, and at least 3 days of hospitalization. An exclusion criterion was having major comorbidities, i.e., other serious or unstable medical conditions that would confound patient’s responses. Participants were recruited among patients of an institute of rheumatology (specialized medical center and teaching hospital). They were contacted by one of three research assistants on the medical ward 1 day after admission.

The data were collected using a diary approach. This approach to data collection can be regarded a special case of a longitudinal design (for details see also [39]). A classical longitudinal study consists of measurements repeated over longer time intervals since its aim is to observe processes which require some time to develop and produce a noticeable change. A diary approach, on the other hand, is focused on shorter time intervals, the longest of which covers 1 day, and on variables that can fluctuate and affect one another within such periods. Thus, since these designs bring different information about a given phenomenon, they are rather complementary than contradictory. A diary approach allows to catch micro-changes that may—as time goes by—result in macro-changes visible in a longitudinal design. In the context of chronic pain, a longitudinal design is suitable when trajectories of psychological adaptation as well as long-lasting functional changes are to be detected and analyzed. However, when the main interest is day-by-day coping with a currently experienced pain level, a diary approach, adopted in this study, is more relevant.

After obtaining informed consent, participants received an envelope containing detailed instructions and three other envelopes, signed with the personal code and the names of the three consecutive days of the study (Tuesday, Wednesday, and Thursday). Due to their representativeness for routine of hospitalization, only weekdays were chosen. Monday and Friday were excluded because they are the usual admission and discharge days. The signed envelopes contained questionnaires to be filled out each evening. The closed envelopes with questionnaires were collected the next day by research assistants also to pace participants’ completion.

### Measures

#### Affect

Affect was measured with the questionnaire proposed by Folkman and Lazarus [40]. It contains 14 adjectives with a seven-point response scale (1 = *not at all*, 7 = *very much so*) evaluating state affect (how a person feels today). Positive affect (PA, *hopeful*, *eager*; *happy*, *pleased*, *relieved*, *exhilarated*, *optimistic*) and negative affect (NA, *worried*, *anxious*, *angry*, *sad*, *disappointed*, *insecure*, *helpless*) subscales were established using exploratory factor analyses, where two factors emerged. Due to the small sample size, congruency coefficients were used to assess factor similarity among measurements instead of confirmatory factor analysis [41]. All the coefficients were calculated with Orthosim 2.1 software by Barrett [42]. The mean value of overall solution congruence was .99 (range .98–.99), which indicates essential identity [43] and therefore can be interpreted as measurement invariance of the obtained two-factor model in the present sample. Cronbach’s alpha coefficients for PA scale were .88, .87, .79 and for NA scale .87, .92, .91 at the three measurement points, respectively.

#### Pain

Daily pain was assessed with a visual analogue scale, i.e., a 10 cm horizontal line anchored by a word description at each end, where a zero (0 cm) meant “no pain at all” and a ten (10 cm) meant “as bad as it could be” [44].

#### Coping

Since there is no well-validated questionnaire that directly measures meaning-focused coping, coping strategies were operationalized on the basis of items derived from the most popular coping questionnaires, such as WCQ [16], CISS [45], and COPE [46], after some rephrasing when necessary. Such procedure has often been adopted in coping research (see for instance [47] or [48] for meaning-focused coping specifically). Then, those items were categorized according to the definitions provided in the introduction into three theoretical categories: emotion-focused, problem-focused, and meaning-focused coping. However, the exploratory factor analyses revealed that items referring to supportive interactions with other people loaded on the separate factor, regardless of their primary allocation within categories. Thus, the coping questionnaire consisted of four subscales describing daily use of strategies with a five-point answering format (1 = *not at all*, 5 = *very much so)*: emotion-focused coping (11 items, e.g., *I‘ve done anything to forget about my own emotions*), problem-focused coping (13 items, e.g., *I’ve wondered how to deal with the problem)*, meaning-focused coping (13 items, e.g., *I’ve told myself that everything that happens in my life makes sense*), and looking for social support (5 items, e.g., *I’ve been looking for support and understanding from others*). Since the latter factor has a different theoretical nature and empirical status (smaller number of items), it was omitted in the study. Finally, stability of these three major factors was reasonably confirmed by a mean overall congruency coefficient value of .90 [34]. Cronbach's alphas were .81, .94, .82, respectively, for emotion-focused coping (EFC), .72, .91, .90, respectively, for problem-focused coping (PFC), and .89, .89, .91, respectively, for meaning-focused coping (MFC).

### Data analyses

Hypotheses were tested using a multilevel approach because of the data’s hierarchical structure, with three daily occasions for each of the 95 participants, resulting in 285 observations in the dataset. Specifically, a two-level model was implemented. Level 1 describes daily affect as a linear function of other repeated variables, that is, pain and coping strategies, which constitutes a *within*-*person* perspective. Level 2 introduced a *between*-*person* perspective assessing how individual differences influence level-1 relations [39]. In order to detect day-to-day change, level-1 predictors were person-centered by subtracting each patient’s individual mean from their daily scores across all observations within a given variable [49]. Those individual means indexed level-2 predictors, again for each variable separately. They were centered around their respective grand means, that is, mean for the whole sample over all measurements, to facilitate interpretation in terms of individual differences. Because of a possible time effect on daily affect, time (centered on the first day) was included into analyses as a covariate. Due to repeated measures, a first-order autoregressive covariance structure was assumed. Intercepts were initially allowed to vary randomly, but since in every model their variation appeared insignificant, they were treated as fixed parameters. Mediation analyses were conducted according to the rules provided by Zhang et al. [50] for lower-level mediation, that is, mediation only for level-1 variables. Thus, the results were controlled for level-2 variances by including the relevant level-2 predictors in each step of mediation testing. The computations were done separately for negative and positive affect. All the analyses were done with IBM SPSS 21.0.

## Results

### Descriptive statistics and missing data analyses

Table 2 presents descriptive statistics and correlations for raw variable scores. The number of missing data did not exceed 5 *%* and followed the pattern of missing completely at random (Little’s MCAR test *χ*^2^ (65) = 82.75, *p* = .07) [51]. As can be seen, autoregressive, that is, day-by-day correlations of the same variables are generally higher than correlations with other variables. Nonetheless, the values still suggest daily fluctuations, and they support the need to separate level-1 and level-2 sources of variance.^1^

**Table 2:** Correlation matrix with means and standard deviations for variables in the study (*N* = 95)

Variable	Pain_1	Pain_2	Pain_3	NA_1	NA_2	NA_3	PA_1	PA_2	PA_3	PFC_1	PFC_2	PFC_3	EFC_1	EFC_2	EFC_3	MFC_1	MFC_2	MFC_3
Pain_1	1.00																	
Pain_2	.75	1.00																
Pain_3	.74	.87	1.00															
NA_1	.35	.22	.20	1.00														
NA_2	.26	.25	.22	.64	1.00													
NA_3	.18	.07	.12	.58	.71	1.00												
PA_1	−.22	−.29	−.25	−.35	−.27	−.16	1.00											
PA_2	−.06	−.21	−.05	−.30	−.30	−.15	.59	1.00										
PA_3	−.10	−.16	−.10	−.24	−.13	−.20	.52	.75	1.00									
PFC_1	.11	.12	.11	−.04	−.04	.01	.05	.01	−.05	1.00								
PFC_2	.28	.26	.29	.03	.03	.00	.13	.29	.24	.69	1.00							
PFC_3	.23	.26	.27	.03	.10	.04	.07	.26	.33	.61	.84	1.00						
EFC_1	.13	.05	.11	−.05	−.07	−.02	.14	.19	.14	.73	.59	.50	1.00					
EFC_2	.25	.20	.25	.00	.04	.00	.26	.33	.31	.47	.69	.54	.66	1.00				
EFC_3	.13	.13	.21	−.04	.04	.03	.18	.36	.44	.38	.61	.69	.53	.68	1.00			
MFC_1	−.09	−.14	−.15	−.21	−.21	−.16	.45	.23	.15	.43	.32	.25	.57	.45	.34	1.00		
MFC_2	.02	.03	.01	−.14	−.11	−.13	.41	.35	.34	.29	.47	.38	.46	.58	.46	.79	1.00	
MFC_3	.03	.04	.08	−.11	−.02	−.09	.31	.33	.42	.13	.41	.46	.31	.48	.64	.57	.78	1.00
M	4.35	4.39	4.56	3.62	3.40	3.27	3.43	3.45	3.46	3.47	3.29	3.31	3.45	3.33	3.38	3.58	3.47	3.50
SD	2.67	2.69	2.68	1.45	1.41	1.42	1.27	1.37	1.40	0.71	0.79	0.72	0.67	0.70	0.61	0.71	0.79	0.77

*Note* All correlations above absolute value of .17 are significant at *p* < .05 (at least). *Pain* pain level, *NA* negative affect, *PA* positive affect, *PFC* problem-focused coping, *EFC* emotion-focused coping, *MFC* meaning-focused coping. Numbers following variable names denote consecutive measurement days

#### **Hypothesis 1**

Relation between affect and meaning-focused coping

The relations between affect and meaning-focused coping were tested in a multilevel approach, preceded by verification of predictors’ interrelations in multiple regression analyses [52], where no multi-collinearity was detected (variance inflation factors below 5). As can be seen in Table 3, in agreement with theoretical expectations, different patterns of relations were observed for NA and PA. Namely, daily level of meaning-focused coping (MFC) was a significant positive predictor for PA, after control for within- and between-person variability in all the other variables, including pain. Such a relation was not noted for NA, where the only significant predictor was level-2 MFC: a lower across-days average intensity of these coping strategies was associated with higher NA. Thus, hypothesis 1 was confirmed. For each additional unit in level-1 MFC on a given day, PA was predicted to be .78 units higher. However, it must be noted that a similar relation was also found for PFC. Finally, after control for coping and separating level-1 and level-2 predictors, there was no significant effect of pain on NA, previously noted in correlational analyses (see Table 2).

**Table 3:** Summary of parameter estimates for multilevel models of affect as a function of pain and coping

Fixed effects	Negative affect				Positive affect			
	Estimate	SE	*t*	*p*	Estimate	SE	*t*	*p*
Intercept	3.69	.16	23.21	<.001	3.43	.13	27.35	<.001
Time	−.24	.08	−2.86	.005	.08	.07	.13	.258
*Level 1*								
Pain	.07	.06	1.24	.218	−.11	.05	−2.28	.024
MFC	−.42	.22	−1.90	.059	.74	.19	4.02	<.001
PFC	−.07	.20	−.36	.723	.35	.17	2.05	.042
EFC	.31	.19	1.61	.109	−.16	.16	−1.02	.308
*Level 2*								
Pain	.08	.06	1.38	.173	−.12	.05	−2.75	.007
MFC	−.62	.27	−2.31	.023	.67	.21	3.26	.002
PFC	.27	.32	.86	.393	.12	.25	.48	.636
EFC	.17	.43	.39	.700	.37	.33	1.12	.267

Covariance parameters (repeated measures)								
	Estimate	SE	*z*	*p*	Estimate	SE	*z*	*p*
Residual	2.00	.25	8.02	<.001	1.25	.15	8.24	<.001
Autocorrelation	.67	.05	15.28	<.001	.66	.05	13.59	<.001

*Note* An autoregressive matrix was used to model the error variance on the dependent variables. Level-1 variables are person-centered. Level-2 variables are sample-centered

*MFC* meaning-focused coping, *PFC* problem-focused coping, *EFC* emotion-focused coping

**Fig. 1:**
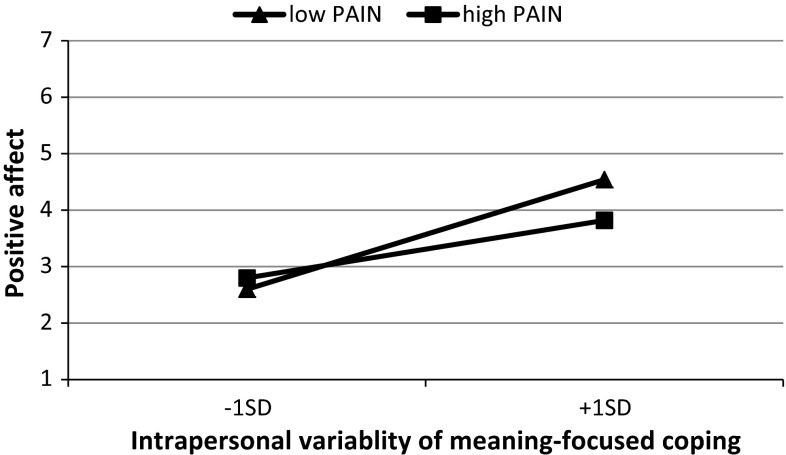
Cross-level interaction: Simple regression slopes for positive affect on intrapersonal variability of meaning-focused coping (level 1) at high and low overall intensity of pain (level 2)

**Fig. 2:**
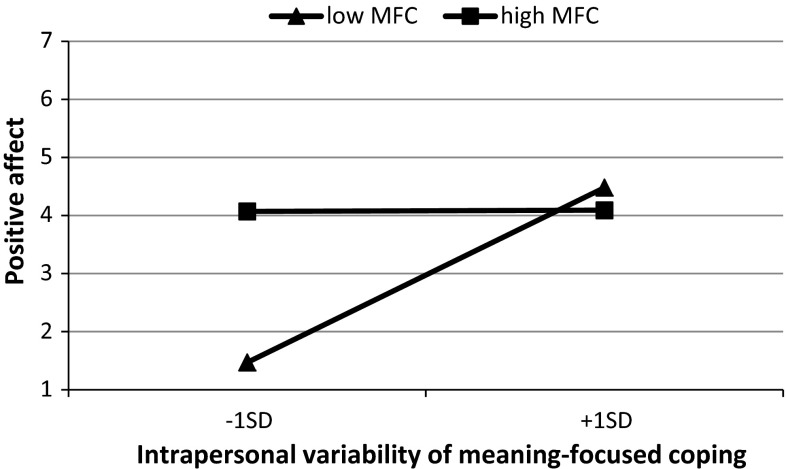
Cross-level interaction: Simple regression slopes for positive affect on intrapersonal variability of meaning-focused coping (level 1) at high and low overall intensity of meaning-focused coping (level 2)

Since PA was related with a higher average pain intensity and higher average MFC, to verify if these predictors moderated within-person MFC-PA relations, possible cross-level interactions were added to the model. Two significant effects were noted. The first one involved between-person differences in pain and in within-person MFC (*B* = −.23, SE = .08, *t* = −2.884, *p* = .004; Fig. 1). Simple slope analyses revealed that for patients experiencing less pain on average, positive affect was more strongly associated with daily level-1 MFC than for patients with a higher average pain level. Also, the positive relation between daily MFC and PA was significant only for patients who reported a lower tendency to use this kind of coping (*B* = −.63, SE = .20, *t* = −2.097, *p* = .038, Fig. 2).

#### **Hypothesis 2**

Meaning-focused coping as a mediator between pain and positive affect

**Fig. 3:**
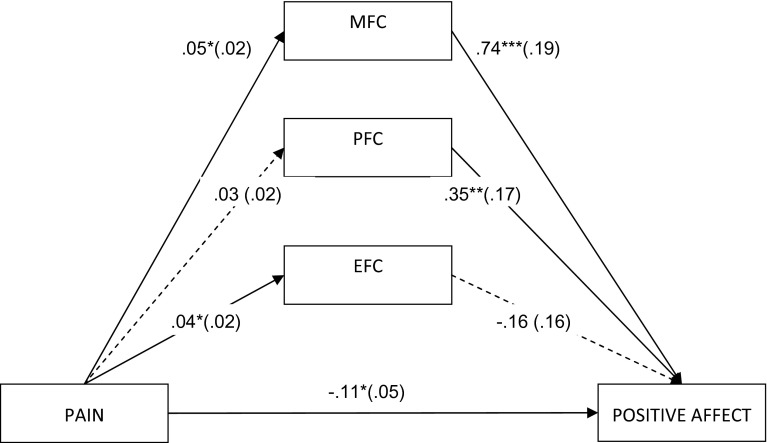
Lower-level multiple mediation model (i.e., level 1 after control for level 2) for relation between pain and positive affect with coping strategies as potential mediators (*MFC* meaning-focused coping, *PFC* problem-focused coping, *EFC* emotion-focused coping). All the presented values are unstandardized. Standard errors are in parenthesis. Dotted lines denote insignificant relations. *p < .01, **p < .05, ***p < .001

The results so far suggest one possible lower-level mediation model, namely a mediation model for pain and PA with meaning-focused and problem-focused coping as potential mediators. However, because we hypothesized a specific effect of meaning-focused coping only, both concurrent strategies (PFC and EFC) were included in the model to be controlled. This model was verified following the classical Baron and Kenny’s [53] steps modified for multilevel data structure to allow interpretation for level-1 mediation only [50]. This entails that all model constituents, that is, the independent variable (pain), mediators (coping strategies), and dependent variable (affect), represent the within-person level after control for between-person variance. The resultant model is shown in Fig. 3. The model uncovers a potential suppressive effect of MFC. There was no significant total effect of pain on PA (*B* = −.07, SE = .05, *t* = −1.53, *p* = .128). Its decomposition shows that this might have been due to a significant protective role of MFC (Sobel’s *z* = 2.07, *p* = .04; indirect effect estimation is .033, 95 % CI [.005, .071]), which suppressed a direct negative influence of pain on PA (*B* = −.11, *p* < .05). Thus, on a given day, a higher pain level was associated with higher MFC, which in turn correlated with higher PA. This confirmed hypothesis 2.

Due to the previously noted significant cross-level interactions, the PA mediation model was additionally verified for moderation regarding paths from mediator to dependent variable. Namely, between-person pain level and MFC might moderate a path from within-person MFC to PA. Thus, finally the indirect effect via MFC appeared significant only for patients who reported lower pain on average (.05; 95 % CI [.01; .11] vs. .02; 95 % CI [.00; .06]), and for patients who reported lower use of MFC on average across all assessment points (.06; 95 % CI [.01; .12] vs. .02; 95 % CI [−.01; .05]).

## Discussion

Although in previous studies the relationship between chronic pain and affect has been intensely explored, the current study is, to our knowledge, the first one that directly examines a role of meaning-focused coping and describes this relation during hospitalization on a day-to-day basis. It was hypothesized that among women hospitalized due to RA, higher values of MFC on a given day would be associated with higher PA, but not with lower NA, and that MFC would suppress the effect of pain on PA. Both these hypotheses were supported. The results can be also interpreted in terms of MFC incremental validity above and beyond PFC and EFC as all analyses were controlled for their possible interrelations. Thus, findings further support a theoretical distinction of MFC from PFC and EFC.

Furthermore, a separation of more stable interindividual characteristics (level 2, between-person) from daily fluctuations (level 1, within-person) revealed limitations to a beneficial role of daily MFC. It seemed to suppress the negative effect of daily pain on PA only when the general level of pain was below sample average, and when this strategy was implemented more in response to the situation than as a general preference. The higher pain intensity is, the more difficult it is to control it through cognitive processes. First, the cognitive functioning itself gets impaired due to pain-related load of limited neuronal resources which in turn impedes self-regulation [54]. Secondly, such pain can be caused by active inflammation, disease progression, or structural changes in joints, all of which are not subject to volitional control [55]. Thus, an implementation of MFC strategies may not be sufficient to sustain daily PA in face of intense pain. Also, using MFC seems to be more beneficial to patients who use it more occasionally than habitually. Keeping in mind that only very few measurements were available, probably too few to comment on the possible patterns, it could be hypothesized that occasional use of MFC may be a more deliberate response to demands of a given day and as such may have been more effective [56]. On the other side, habitual use of MFC may merely reflect personal preferences, independent from situational context. Therefore, some mismatch between more frequent implementation of such strategies and changes in day-by-day pain level may occur.

However, it must be noted that patients who used MFC with an overall higher intensity had a generally higher level of PA (controlled for pain), independent of these coping strategies’ daily variations. Thus, both kinds of use (habitual vs contextual) may be beneficial, but for different persons and probably through different mechanisms. Taken together, it shows an interesting interplay between stable (level-2 "style") and contextual (level-1 "strategy") aspects of coping behavior. Clinically, these findings may contribute to better fit interventions to patients’ needs which are of special importance when effective coping with chronic pain is fundamental for health-related quality of life [57]. More traditional data analyses do not allow for the separation of such effects.

Additionally, there was no relation between NA and pain at any level after control for coping strategies. It may suggest the effectiveness of coping, even if only level-2 MFC appeared to be significant. On the other hand, co-occurrence of negative affect and pain is probably not so obvious as assumed on the basis of findings from cross-sectional studies, where between- and within-person variance is not systematically separated [58]. In studies that take into account a hierarchical data structure, the aforementioned relationship has already been noted as insignificant, especially when a moderate or lower pain level was considered. This was also the case here as the sample pain mean was below five on the ten-point pain scale. For instance, Hamilton et al. [59] did not obtain the prospective effect of pain on NA for women with rheumatoid arthritis assessed in weekly intervals. The level of pain as well as a baseline zero-order correlation between pain and NA in that group was similar to the one reported in the current study. Using a within-day perspective, such lack of significant relationships between pain and NA was noted by Newth and Delongis [60], as well. This was also the case in the prototypical study differentiating individual and contextual influences in relations between daily hassles, mood, and chronic pain by Affleck et al. [61]. Nonetheless, these null findings can be misleading [59] because plenty of level-2 moderators of the pain and NA relationship have already been reported, including a history of depressive episodes, vulnerability or pain acceptance [62–64]. Still, this may indicate that a debilitating effect of pain on state affect is not necessarily true for every RA patient (see also [65] for comparison).

However, the current study has limitations that should be kept in mind when discussing the results. Although a diary approach was implemented, the present design consisted only of few measurements, which was determined mainly by an expected short duration of the participants’ hospitalization, but may result in insufficient statistical power. Alternatively, such an approach is more reliable than a cross-sectional study. Still, the question arises how this might influence the findings. When the raw correlations were inspected carefully, we noticed that all coefficients were generally weaker for the third day of the study, compared to relations noted for the previous days. Two explanations are possible. First, this may be an artifact due to the testing procedure, an effect already observed in other dairy studies [66]. However, quite interestingly, this effect would address only correlations among indicators of different constructs as this drop was not noted for autocorrelations among indicators of the same construct over time. Also, stable mean and SD values would not support this methodological argument. Thus, another explanation should be considered: Weakening of correlations can be a sign of an adaptation process and because of it results should be interpreted mainly in the context of the first days of hospitalization. Moreover, the correlative character of the study design makes all the interferences only probabilistic. Data collection was also restricted to women. Accordingly, findings may also be valid only for women with RA, as in previous research significant gender differences are systematically noted with regard to pain intensity and affect [67, 68]. It must be noted, however, that the first days after admission are probably the most challenging for patients, and that a majority of those diagnosed with RA are women, so the clinical value of the obtained results seems promising.

Finally, a wide range of patients’ age can be perceived as both a weakness and a strength of the study. Older age among RA patients is connected with higher comorbidity [69], which was not sufficiently included in the study, also due to the fact that at the time of the study, RA was the patients’ only major health concern. On the other hand, age appeared to be normally distributed and unrelated to pain, affect, and coping so there is no evidence that older patients in our sample provided any substantially different data on these variables. Thus, as older adults (≥65 years) have rarely participated in the studies regarding coping with RA, our findings may suggest that age itself is not a determining factor underlying patient’s actual functioning (see also [70, 71]), which should stimulate further research in this area.

To sum up, daily meaning-focused coping was found to suppress the negative effect of daily pain on positive affect. Advanced methodological and statistical approaches allow to separate within- from between-person sources of variance and to determine the limits of the aforementioned effect. Also, as far as we know, it is the only diary study of RA patients during hospitalization. As such, it has a strong clinical relevance regarding the high hospitalization rate among this group of patients, who cite pain as one of the leading causes of lowered quality of life [7].

## Footnotes

1 Additionally, because of the wide age range it was checked whether age was significantly related to any of the variables in the study. No significant correlations emerged. The same was true for years since diagnosis, with the only exception of negative affect at the second measurement (Spearman’s *ρ* = .232, *p* = .02). Also, age and time since diagnosis were weakly positively correlated (Spearman’s *ρ* = .230, *p* = .02).
